# Cost-Effectiveness of Technology-Assisted Case Management in Low-Income, Rural Adults with Type 2 Diabetes

**DOI:** 10.1089/heq.2020.0134

**Published:** 2021-07-26

**Authors:** Leonard E. Egede, Clara E. Dismuke, Rebekah J. Walker, Joni S. Williams, Christian Eiler

**Affiliations:** ^1^ Division of General Internal Medicine, Department of Medicine, Medical College of Wisconsin, Milwaukee, Wisconsin, USA.; ^2^ Center for Advancing Population Science, Department of Medicine, Medical College of Wisconsin, Milwaukee, Wisconsin, USA.; ^3^ Health Economics Resource Center (HERC), VA Palo Alto Health Care System, Palo Alto, California, USA.

**Keywords:** cost-effectiveness, low-income, rural, technology, case management, diabetes

## Abstract

**Objective:** The objective of this study was to examine whether delivering technology-assisted case management (TACM) with medication titration by nurses under physician supervision is cost effective compared with usual care (standard office procedures) in low-income rural adults with type 2 diabetes.

**Methods:** One hundred and thirteen low-income, rural adults with type 2 diabetes and hemoglobin A1c (HbA1c) ≥8%, were randomized to a TACM intervention or usual care. Effectiveness was measured as differences in HbA1c between the TACM and usual care groups at 6 months. Total cost per patient included intervention or usual care cost, medical care cost, and income loss associated with lost workdays. The total cost per patient and HbA1c were used to estimate a joint distribution of incremental cost and incremental effect of TACM compared with usual care. Incremental cost-effectiveness ratios (ICERs) were estimated to summarize the cost-effectiveness of the TACM intervention relative to usual care to decrease HbA1c by 1%.

**Results:** Costs due to intervention, primary care, other health care, emergency room visits, and workdays missed showed statistically significant differences between the groups (usual care $1,360.49 vs. TACM $5,379.60, *p*=0.004), with an absolute cost difference of $4,019.11. Based on the intervention cost per patient and the change in HbA1c, the median bootstrapped ICERs was estimated to be $6,299.04 (standard error=731.71) per 1% decrease in HbA1c.

**Conclusion:** Based on these results, a 1% decrease in HbA1c can be obtained with the TACM intervention at an approximate cost of $6,300; therefore, it is a cost-effective option for treating vulnerable populations of adults with type 2 diabetes.

## Introduction

More than 34 million individuals of all ages are estimated to have diabetes, of which 90–95% have type 2 diabetes.^1^ Diabetes is more prevalent in racial/ethnic minorities and rural residents and varies significantly by socioeconomic status.^1,2^ In addition, estimates show total costs for diagnosed diabetes to be $327 billion, accounting for $237 billion in direct medical costs, and $90 billion in lost productivity, which suggests a 26% increase in costs related to diabetes since 2012.^3^

Hemoglobin A1c (HbA1c) is the standard clinical measure used to capture average blood glucose levels for the prior 3 months, and an important measure for preventing diabetes-related complications.^1^ Complications of diabetes are both detrimental to quality of life and account for the majority of long-term costs for managing type 2 diabetes.^4^

Nurse case management has been shown to have a positive impact on treatment adherence, patient satisfaction, clinical measurements, self-management, and quality of life in chronic disease.^5^ Nurse case management has been demonstrated to enhance quality of care, improve behavioral and clinical outcomes, and reduce costs in adults with type 2 diabetes,^6,7^ especially when used in combination with technology^8–12^ Several randomized controlled trials have shown nurse case management to be an effective strategy for improving glycemic control.^6,13–18^ A recent randomized controlled trial assessing technology-assisted case management (TACM) with medication titration by nurses in low-income, rural adults found the intervention to be efficacious in improving glycemic control.^19,20^ Compared with usual care, or standard office-based practice, individuals who received the TACM intervention had a significantly lower HbA1c at 6 months and a faster rate of decline.^20^

A limiting factor to implementing technology-supported case management and titration by nurses under supervision at a large scale is lack of cost-effectiveness data to support reimbursement for care.^21–24^ Authors have noted the importance of conducting cost evaluations across different populations and intervention types as the generalizability for complex programs is highly dependent on how care was organized.^25^ However, results are still pending for a number of cost-effectiveness trials on case management or technology-delivered diabetes care.^6,26–29^

Current studies have found case management interventions to be costlier than controls to obtain the same outcome, especially in vulnerable populations.^30–33^ In one study, a culturally modified stepped care case management approach found no difference in hospital expenditures during the study, however, when authors used a simulation model that accounted for life expectancy, quality-adjusted life years (QALYs), and complications over a 40-year horizon, the cost-effectiveness was significant.^34,35^ Two studies, including a care coordination/home telehealth program delivered to veterans with diabetes, and an integrated diabetes management program provided by a network of general practitioners, found favorable incremental cost-effectiveness ratios (ICER), however, some were as high as $60,000 per QALY.^36,37^

Given the inconsistent results of prior cost-effectiveness studies and the possible benefit of the TACM intervention for a particularly vulnerable group (low-income, rural adults), the objective of this study was to examine whether the TACM intervention (TACM with medication titration by nurses under physician supervision) is cost-effective compared with usual care in a low-income rural adult population.

## Methods

### Study design and participants

TACM was a randomized control trial, which examined the effectiveness of the FORA 2-in-1 telehealth system in controlling HbA1c at 6 months. Details of the protocol for the trial have been documented elsewhere and are briefly outlined below.^19,20^ Participants were low-income, rural adults (age ≥18) with type 2 diabetes receiving care at the clinics of a Federally Qualified Health Center in South Carolina. Inclusion criteria included diagnosis of type 2 diabetes, an HbA1c ≥8.0%, a working landline telephone, ability to speak English, and willingness to use the FORA 2-in-1 telemedicine device. Exclusion criteria included mental confusion examined at initial interview, participation in other diabetes control trials, presence of alcohol abuse/dependence, pregnancy or lactation, or a life expectancy of <6 months.

### Study setting and randomization

In the randomized controlled trial, participants were recruited from eight community-based adult medicine primary care practices within the Franklin C. Fetter Health Center, a Federally Qualified Health Center in South Carolina, based on International Classification of Diseases-9 codes consistent with a diagnosis of type 2 diabetes from clinic billing data and laboratory data or referrals from physicians, other clinic staff such as nurses, or patients themselves. Letters of invitation signed by the patients' primary care providers were mailed to patients, and IRB-approved recruitment flyers were posted in prominent locations within the study clinics.

After verifying inclusion and exclusion criteria, and completing informed consent, the nurse randomized each participant (1:1) to either TACM or usual care. Randomization was performed in waves where ∼50 participants were randomized every 6 months. The randomization sequence was web based, and computer generated and was accessible to the nurse case manager, who delivered the intervention and was not blinded to randomization assignment. The research assistants, blinded to treatment assignments, collected primary data on the participants. Participants and treating physicians were not blinded because of the nature of the intervention. All study procedures were approved by the local Institutional Review Board. The study is registered at ClinicalTrials.gov (NCT01373489).

One hundred and thirteen patients were randomized to either usual care or the intervention group. Study attrition was 28 individuals due to either withdrawing consent or loss to follow-up for a per-protocol intervention group of 41 patients and per-protocol usual care group of 44. Those who withdrew or were lost to follow-up were similar in characteristics (i.e., demographics, seen at one of the eight community-based adult medicine primary care practices within the Franklin C. Fetter Health Center, diagnosed with type 2 diabetes) to those who participated in the study.

### TACM intervention description

TACM is a high-intensity form of case management that capitalizes on information technology to link a case manager to individuals with poorly controlled diabetes in real time. Individuals randomized to the TACM group received the FORA 2-in-1 Telehealth System for diabetes management. One nurse case manager delivered the intervention, which began with the case manager teaching the patients how to use the FORA device and asking them to provide daily blood glucose and blood pressure measurements. In addition, the patients were taught how to problem solve around the daily readings, which were linked to the nurse case manager in real time. Based on FORA measurements and evidence-based treatment algorithms for diabetes and hypertension approved by the primary care providers at Franklin C. Fetter, the nurse case manager made medication adjustments weekly (for patients on insulin) or biweekly (for patients on oral agents) under the supervision of the study physicians, an internist, and endocrinologist.

### Usual care

Those randomized to usual care received the current standard of care at the study clinics, where the providers were responsible for developing treatment plans in collaboration with the patients. The clinic providers included the physicians, nurse practitioners, or physician assistants normally caring for the patients. Contact between the scheduled visits at the study clinics for follow-up and/or abnormal results were patient initiated and addressed by the clinic nurses. In addition, the frequency of scheduled visits was patient initiated and coordinated with the clinic staff. Finally, all usual care visits were in-person per normal routine at the study clinics.

### Clinical outcome

The primary clinical outcome was glycemic control (HbA1c) at 6 months follow-up. HbA1c was measured through blood specimens drawn at three times (at baseline, at 3 months follow-up, and at 6 months follow-up). It has been previously documented that TACM significantly improved glycemic control compared with the usual care group at the 6 months follow-up.^20^

### Study cost

Intervention, health care, and patient (lost wages) costs were obtained based on a societal perspective, which included health care utilization and patient costs which was recommended by the U.S. Public Health Service Panel on Cost-Effectiveness in Health and Medicine and Pyne et al.^38^ All costs were adjusted for inflation using the U.S. Department of Labor Inflation Calculator^39^ to reflect 2021-dollar values.

### Intervention cost

Cost for institutional overhead ($1,454.20) and staffing of a nurse ($70,016.88) were applied to the treatment group per patient. Overhead cost was based on renting a 150 sq. ft. office at the rate of $1.62 a sq. ft. for 6 months. Distributing institutional overhead cost over the 41 patients, resulted in a mean overhead cost of $35.47 per patient. Distributing the nurse staffing cost over the 41 patients, resulted in a mean nursing cost of $1,707.73 per patient. The FORA device, test strips, lancing device, and web system had a mean cost of $113.10 dollars per patient. Summing the institutional, nursing, and FORA supplies costs per patient resulted in a total cost of $1,856.30 per patient. After adjustment for inflation, these costs resulted in a 2021-dollar values of $1,988.76 per patient.^39^

### Health care cost

Costs for medical treatment were estimated using self-reported health care utilization amounts and Medical Expenditure Panel Survey (MEPS) mean expenditures for each type of utilization and adjusted to 2021-dollar values using U.S. Department of Labor Inflation Calculator.^39^

### Lost income

Wages were self-reported by participants in intervals. To estimate lost wages due to illness, an interval regression was used to estimate wages of individuals, adjusting for patient age, gender, ethnicity, educational level, and self-reported health status. Mean estimated wages were then divided by the number of annual working days (260) to get a mean wage per working day. The mean value was then multiplied by self-reported lost days of work due to illness, to estimate total lost income per patient due to illness. Mean estimated wages were reported in 2012 dollars by patients so they were adjusted for inflation to 2021-dollar values using the U.S. Department of Labor Inflation Calculator.^39^

### Statistical analyses

All analyses were conducted using Stata/SE 15 and R (StataCorp) (R Core Team). First, initial comparisons were conducted to investigate differences in sample characteristics between the control and TACM group using *χ*^2^ for categorical and t-tests for continuous variables. Second, clinical outcomes and costs were calculated and compared using t-tests. Finally, analyses were conducted to estimate the cost-effectiveness of TACM, following methodology established by a similar previous study.^38^ The main difference between this and the Pyne study was that the Pyne study used QALYs, whereas this study used changes in HbA1c for the main effectiveness outcomes.

Participants randomized per protocol were used for the analysis, with statistical significance considered at *p*<0.05. ICERs were estimated based on a standard methodology to summarize the cost-effectiveness of a health care intervention relative to a control.^38^ ICERs were calculated as the difference in cost between the intervention (TACM) and the usual care group, divided by the difference in outcomes (HbA1c) between the intervention (TACM) and the usual care group. Total cost per patient included intervention or usual care cost, medical care cost, and income associated with lost workdays. The total cost per patient and HbA1c were used to estimate a joint distribution of incremental cost and incremental effect of TACM, compared with usual care, as well as a cost-effectiveness acceptability curve for a change in HbA1c.

A limitation of the ICER is a possible zero difference between the intervention and usual care group in the outcome will cause the ICER to statistically approach infinity. Also, typical standard error estimation methods do not apply to cost-effectiveness ratios due to cost and effectiveness estimates rarely being independent, therefore a nonparametric distribution of errors was assumed. Results were generated using a nonparametrically bootstrapped sample with replacement of 1000 observations to provide mean and standard deviation estimates, along with confidence intervals of incremental intervention costs and incremental effectiveness measures (HbA1c).^38^ These estimates were then used to generate scatter plots of incremental costs and differences in HbA1cs, and a cost-effectiveness acceptability curve for a change in HbA1c. The cost–acceptability curve is a visual to illustrate if an intervention offers “good” value for money.^40^

Thresholds for a patient's willingness to pay have been developed by the World Health Organization based upon a country's per-capita gross domestic product.^41^ The ICER is compared with this monetary threshold, which represents the maximum amount that the decision maker (in this case, the patient) is willing to pay for health effects (in this case, a 1% drop in HbA1c). The intervention is deemed cost effective if the ICER falls below this threshold and not cost effective otherwise.

## Results

Table 1 provides demographic information for the per protocol participants in the randomized controlled trial. Participants were middle-aged (mean age=55.33), primarily non-Hispanic Black, and female, with low income (mean individual income=$14,652.02 after inflation to 2021 dollars) and worked on average 10.5 h per week. There were no statistically significant differences in age, gender, race/ethnicity, education, marital status, income, or insurance status between the usual care group and intervention groups at baseline.

**Table 1.** Demographics by Treatment Group of Per-Protocol Participants

	Usual care	TACM	*p*
	*n*=44	*n*=41	
Age, mean (SD)	55.05 (±10.42)	55.63 (±11.11)	0.80
Gender			0.66
Female	38 (86.36%)	34 (82.93%)	
Male	6 (13.64%)	7 (17.07%)	
Race			0.13
White	5 (11.36%)	7 (17.07%)	
Black	39 (88.64%)	31 (75.61%)	
Other	0 (0.00%)	3 (7.32%)	
Education			0.12
Less than HS	12 (27.27%)	4 (9.76%)	
HS diploma	19 (43.18%)	22 (53.66%)	
More than HS	13 (29.55%)	15 (36.59%)	
Marital status			0.99
Married	14 (68.18%)	13 (68.29%)	
Not married	30 (31.82%)	28 (31.71%)	
Income			0.87
$<10,000	13 (29.55%)	15 (36.59%)	
$<15,000	14 (31.82%)	12 (29.27%)	
$<25,000	12 (27.27%)	11 (26.83%)	
$25,000+	5 (11.36%)	3 (7.32%)	
Insurance			0.23
Insured	19 (43.18%)	23 (56.10%)	
Not insured	25 (56.82%)	18 (43.90%)	
Employment			
mean (SD) hours worked per week	12.50 (±2.47)	8.44 (±2.35)	0.24

HS, high school; SD, standard deviation; TACM, technology-assisted case management.

Table 2 provides information on the clinical outcomes, and Table 3 provides information on the cost outcomes. Although there were no differences between groups in HbA1c at baseline, HbA1c was significantly different statistically at 6-month follow-up for the TACM intervention, compared with the usual care group (*p*=0.04), with an absolute difference of nearly 1%. Based on calculations of cost due to intervention, primary care, other health care, emergency room visits, and workdays missed, there were statistically significant medical care cost differences between the groups (*p*=0.004). The usual care group had a mean medical care cost of $1,360.49 (standard deviation [SD]=1675.78), whereas the intervention group had a mean medical care cost of $5,379.60 (SD=8442.72), with an absolute cost difference of $4,019.11.

**Table 2.** Changes in Clinical Outcome (Hemoglobin A1c) by Treatment Group

HbA1c	Usual care	TACM	*p*
	Mean (SD)	Mean (SD)	
Baseline	10.31 (2.22)	9.98 (1.75)	0.45
6-month	10.05 (2.66)	9.00 (1.85)	0.04^a^
Mean change	−0.27 (2.16)	−0.98 (2.06)	0.13
Median change	0 (−0.70, 0.20)	−0.7 (−1.43, −0.27)	

^a^ Statistically significant at *p*<0.05.

HbA1c, hemoglobin A1c.

**Table 3.** Costs by Treatment Group

Costs	Usual care	TACM	
	Mean (SD)	Mean (SD)	
Intervention	—	$1,998.76	
Primary care	$423.91 (337.65)	$ $373.29 (346.75)	
Other health care	$848.74 (1491.40)	$2,240.20 (4879.90)	
ER visits	—	$628.02 (3685.26)	
Workdays missed	$87.84 (313.82)	$149.33 (426.21)	
*Total*	$1,360.49 (1675.78)	$5,379.60 (8442.72)	*p*=0.004
*Absolute difference*	$4,019.11		

ER, emergency room.

**FIG. 1.**
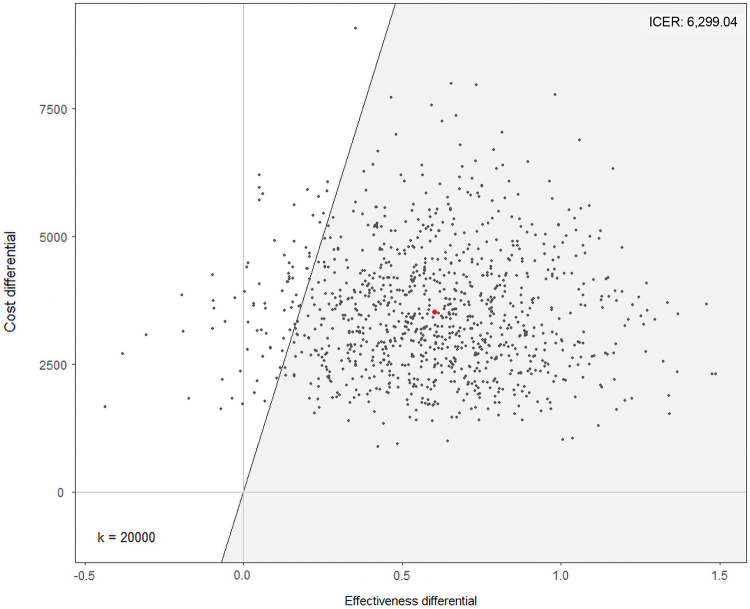
Cost-effectiveness plane for TACM versus usual care, with *bolded red dot* indicating the ICER after 1000 bootstrap iterations. ICER, incremental cost-effectiveness ratio; TACM, technology-assisted case management.

**FIG. 2.**
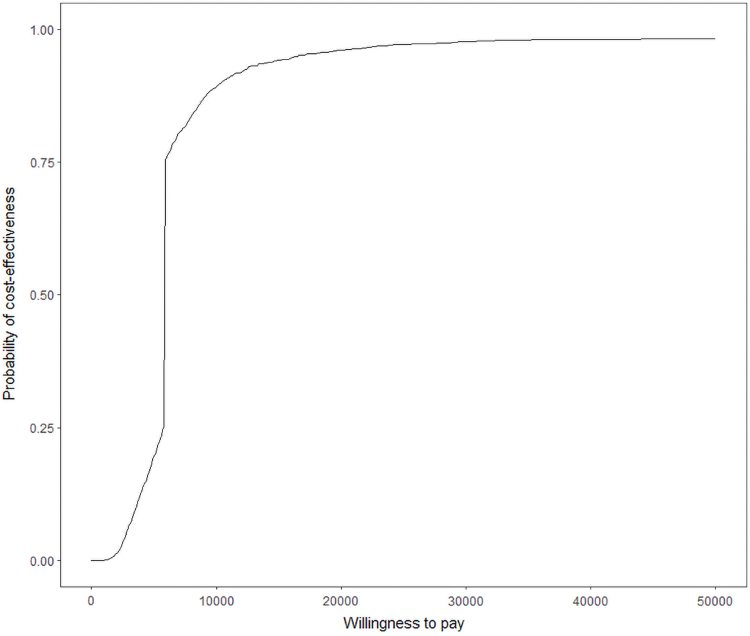
Cost-effectiveness acceptability curve indicating the probability of cost-effectiveness given a willingness to pay level for the TACM intervention.

Based on the intervention cost per patient and the change in HbA1c, the median bootstrapped ICERs was estimated to be $6,299.04 (standard error=731.71) per 1% decrease in HbA1c. A scatter plot analysis of incremental cost and difference in HbA1c can be seen in Figure 1, and a cost–acceptability curve for change in HbA1c can be seen in Figure 2, both using methodology suggested by Pyne et al.^38^ The scatter plot analysis shows a bolded red dot for the ICER after 1000 bootstrap iterations, and a diagonal line for the point at which each cost differential and effectiveness differential would be considered cost effective. The ICER and the majority of the bootstrapped iterations are in the top right quadrant of the graph indicating the intervention is more costly, but also more effective than the control, and are within the range of cost-effectiveness based on the diagonal line indicating willingness to pay. For this study, the cost–acceptability curve was set from the perspective of the patient and indicates the probability of a range of costs associated with the intervention, medical visit costs, and lost income combined falling below cost-effectiveness ratio thresholds for a 1% drop in HbA1c. Based on the cost–acceptability curve, a cost of $6,299.04 per 1% decrease in HbA1c makes the TACM intervention cost effective.

## Discussion

This study provides a cost-effectiveness analysis for a TACM program delivered to a low-income, rural adult population with type 2 diabetes. We found a cost difference of just over $4,000, and an ICER of just over $6,300 for a 1% decrease in HbA1c. Particularly given that technology costs have decreased since the time of the study, results indicate high cost-effectiveness for achieving a 1% decrease in HbA1c. Standardized cost savings for 1% change in HbA1c range from $1,000–$4,000 per person per year depending on their current glycemic control and number of comorbidities.^42,43^ Although this study does not calculate QALYs, this ICER could be considered a minimal cost increase to achieve the expected societal impact of $40,439/QALY found in previous studies to result from decreasing the complications that result from uncontrolled HbA1c.^44^ In addition, costs resulting from type 2 diabetes complications are known to accrue over a long time frame, with estimates of cost-effectiveness being likely over the long term with ICERs below $100,000.^45^

This study adds significantly to the literature by providing a cost-effectiveness analysis of an intervention that can be implemented in low resourced facilities to improve glycemic control, thereby addressing health equity concerns. The study population is vulnerable on a variety of fronts, being focused on rural and low-income patients, and having a high representation of non-Hispanic Black patients. Having been tested in a primarily rural population, this study highlights an intervention that can be implemented to reach populations that may have lower access to care.

As TACM used technology that required only a phone line, the intervention may be less likely to exacerbate the digital divide than interventions requiring more sophisticated technology or requiring broadband access. In addition, as this study targeted a low-income population, the cost-effectiveness of the intervention suggests health systems could implement TACM without reliance on significant costs to patients. The mean income of the study population was quite low compared with the U.S. population mean, however, given this mean is individual income, it is consistent with earnings of $7.04 per hour, which is close to the $7.25 per hour federal minimum wage. Based on results of this study, TACM is both clinically effective and cost effective, providing an evidence-based option for health systems and health insurers to implement into clinical settings serving vulnerable patient populations.

ICERs for prior analyses of case management programs ranged from ∼$7,000 to over $60,000 depending on the details of case management and the population. A culturally modified stepped care case management approach found ICERs that ranged from $10,141 to $69,587 for a 40-year horizon.^35^ Over a shorter time frame and considered in relation to QALYs, a care coordination/home telehealth program delivered to veterans with type 2 diabetes found an average 1-year ICER of $60,941 per QALY.^36^ ICERs for an integrated diabetes management program provided by a network of general practitioners ranged from $8,108 per life year and $9,730 per year increase in quality-adjusted life expectancy.^37^ Finally, large-scale care management, such as the patient-centered medical home models, were found to offer savings of $7,898 per QALY when focused only on higher risk populations.^33^ The results of this study suggest the TACM intervention is a cost-effective alternative to usual care, and is similar, if not more cost effective than other case management options.

From a clinical perspective, the TACM intervention provides a practical and sustainable system of diabetes management that can help low-income and rural patients with type 2 diabetes achieve treatment goals that can be sustained over time. One benefit of nurse case management allows nurses to work independently to provide high-quality and cost-effective care.^7^ TACM demonstrates the successful utilization of a solitary nurse to manage a high-volume case load, while simultaneously performing high-intensity case management and maintaining and monitoring patient safety. TACM allows for frequent communication between a nurse and the patients, which has been shown to influence outcomes.^46^ Finally, as telemonitoring shows promising results in improving diabetes-related outcomes,^8^ the cost-effectiveness of the TACM intervention suggests combining case management with technology can extend the reach of care, while providing improved outcomes for the increased cost. While the cost of the TACM intervention was higher, the improvement in HbA1c was such that the intervention was more cost effective at reaching a 1% decrease. As this level of improvement in glycemic control has been associated with better outcomes and fewer complications,^47,48^ this study suggests cost-effectiveness at 6 months and possible continued cost-effectiveness in the future.

Although strengthened by the randomized controlled design of the initial trial and the focus on a particularly vulnerable population to confirm cost-effectiveness with this group, this study has some limitations. First, participants were required to have an HbA1c above 8%, and as such cost-effectiveness may be specific to a population with uncontrolled diabetes. Indeed, an intervention targeted to a higher HbA1c range where larger changes in HbA1c are possible, are more likely found to be cost effective than those targeted to a HbA1c range, which is closer to the normal. This is because smaller changes in the clinical outcome will cause the ICER to become much larger, approaching statistical infinity when there is a zero or near-zero difference between the clinical outcome of the intervention and usual care group. Alternatively, if participants had been required to have an HbA1c above 9, it is very likely the ICER would be smaller, and therefore even more acceptable under cost–acceptability scenarios.

Second, long-term costs are not captured as utilization extends only to the end of the trial. Given these costs are often major drivers of the burden of diabetes, the ICER could be more cost effective if considered over a longer time horizon.

Third, the intervention costs were based on one medical center located in the southeastern United States and may not reflect the national costs of institutional overhead and salaries. Similarly, patient lost wages are geographic in nature and may not reflect national patient expected lost wages. However, health services utilization costs were taken from MEPS, which is a national sample of health care expenditures for the U.S. population.

Fourth, following similar studies, health care utilization was self-reported, however given possible recall bias this may be under-represented. Given the randomized nature of the study, there is no expectation that either arm would have differential recall, so additional utilization would not be expected to impact cost-effectiveness calculations.

Finally, patients in the intervention group were asked to provide daily blood glucose and blood pressure measurements, which may have influenced their perspectives of or satisfaction with the intervention. Several strategies were implemented to increase adherence to testing, such as stressing the importance of testing and uploading daily readings with the patients, and providing reminder calls to the patients. Satisfaction with the intervention was high and would not be expected to influence cost-effectiveness within this per-protocol sample, however, if implemented with less frequent testing, efficacy of the intervention may be lower.

In conclusion, given the need for cost-effectiveness evidence to support reimbursement of case management, this study offers important information regarding the possible return on investment for patients with type 2 diabetes to achieve a 1% decrease in HbA1c from a TACM program delivered by nurses with physician supervision of medication titration. Based on these results, a 1% decrease in HbA1c can be obtained with the TACM intervention at an approximate cost of $6,300; therefore, it is a cost-effective option for treating vulnerable populations of adults with type 2 diabetes.

## Abbreviations Used

ER: emergency room
HbA1c: hemoglobin A1c
HS: high school
ICER: incremental cost-effectiveness ratio
MEPS: Medical Expenditure Panel Survey
QALY: quality-adjusted life year
SD: standard deviation
TACM: technology-assisted case management

## References

[1] Centers for Disease Control and Prevention. National Diabetes Statistics Report, 2020. Atlanta, GA: Centers for Disease Control and Prevention, U.S. Dept of Health and Human Services, 2020

[2] Rutledge SA, Masalovich S, Blacher RJ, et al. Diabetes self-management education programs in nonmetropolitan counties—United States, 2016. MMWR Surveill Summ. 2017;66(No. SS-10):1–6 10.15585/mmwr.ss6610a1 PMC5829897 28448482

[3] American Diabetes Association. Economic costs of diabetes in the U.S. in 2017. Diabetes Care. 2018;41:917–928 29567642 10.2337/dci18-0007 PMC5911784

[4] Liebl A, Khunti K, Orozco-Beltran D, et al. Health economic evaluation of type 2 diabetes mellitus: a clinical practice focused review. Clin Med Insights Endocrinol Diabetes. 2015;8:13–19 25861233 10.4137/CMED.S20906 PMC4374638

[5] Sutherland D, Hayter M. Structured review: evaluating the effectiveness of nurse case managers in improving health outcomes in three major chronic diseases. J Clin Nurs. 2009;18:2978–2992 19747197 10.1111/j.1365-2702.2009.02900.x

[6] Stuckey HL, Dellasega C, Graber NJ, et al. Diabetes nurse case management and motivational interviewing for change (DYNAMIC): study design and baseline characteristics in the chronic care model for type 2 diabetes. Contemp Clin Trials. 2009;30:366–374 19328244 10.1016/j.cct.2009.03.002 PMC2740652

[7] Ritter J, Fralic MF, Tonges MC, et al. Redesigned nursing practice: a case management model for critical care. Nurs Clin North Am. 1992;27:119–128 1545984

[8] Flodgren G, Rachas A, Farmer AJ, et al. Interactive telemedicine; effects on professional practice and health care outcomes. Cochrane Database Syst Rev. 2015;9:CD002098 10.1002/14651858.CD002098.pub2 PMC6473731 26343551

[9] Bellazzi R, Arcelloni M, Bensa G, et al. Design, methods, and evaluation directions of a multi-access service for the management of diabetes mellitus patients. Diabetes Technol Ther. 2003;5:621–629 14511417 10.1089/152091503322250640

[10] Quinn CC, Clough SS, Minor JM, et al. WellDoc mobile diabetes management randomized controlled trial: change in clinical and behavioral outcomes. Diabetes Technol Ther. 2008;10:160–168 18473689 10.1089/dia.2008.0283

[11] McMahon GT, Gomes HE, Hickson Hohne S, et al. Web-based care management in patients with poorly controlled diabetes. Diabetes Care. 2005;28:1624–1629 15983311 10.2337/diacare.28.7.1624 PMC1262644

[12] Stone RA, Rao RH, Sevick MA, et al. Active care management supported by home telemonitoring in veterans with type 2 diabetes: the DiaTel randomized controlled trial. Diabetes Care. 2010;33:478–484 20009091 10.2337/dc09-1012 PMC2827492

[13] Aubert RE, Herman WH, Waters J, et al. Nurse case management to improve glycemic control in diabetic patients in a health maintenance organization. A randomized, controlled trial. Ann Intern Med. 1998;129:605–612 9786807 10.7326/0003-4819-129-8-199810150-00004

[14] Piette JD, Weinberger M, Kraemer FB, et al. Impact of automated calls with nurse follow-up on diabetes treatment outcomes in a Department of Veterans Affairs Health Care System: a randomized controlled trial. Diabetes Care. 2001;24:202–208 11213866 10.2337/diacare.24.2.202

[15] Shea S, Weinstock RS, Teresi JA, et al. A randomized trial comparing telemedicine case management with usual care in older, ethnically diverse, medically underserved patients with diabetes mellitus: 5 year results of the IDEATel study. J Am Med Inform Assoc. 2009;16:446–456 19390093 10.1197/jamia.M3157 PMC2705246

[16] Welch G, Garb J, Zagarins S, et al. Nurse diabetes case management interventions and blood glucose control: results of a meta-analysis. Diabetes Res Clin Pract. 2010;88:1–6 20116879 10.1016/j.diabres.2009.12.026

[17] Curtis J, Lipke S, Effland S, et al. Effectiveness and safety of medication adjustments by nurse case managers to control hyperglycemia. Diabetes Educ. 2009;35:851–856 19713556 10.1177/0145721709343677

[18] Kwon HS, Cho JH, Kim HS, et al. Development of web-based diabetic patient management system using short message service (SMS). Diabetes Res Clin Pract. 2004;66(Suppl. 1):S133–S137 15563964 10.1016/j.diabres.2003.10.028

[19] Egede LE, Strom JL, Fernandes J, et al. Effectiveness of technology-assisted case management in low income adults with type 2 diabetes (TACM-DM): study protocol for a randomized controlled trial. Trials. 2011;12:231 22014122 10.1186/1745-6215-12-231 PMC3219699

[20] Egede LE, Williams JS, Voronca DC, et al. Randomized controlled trial of technology-assisted case management in low income adults with type 2 diabetes. Diabetes Technol Ther. 2017;19:476–482 28581821 10.1089/dia.2017.0006 PMC13170820

[21] Valentine WJ, Curtis BH, Pollock RF, et al. Is the current standard of care leading to cost-effective outcomes for patients with type 2 diabetes requiring insulin? A long-term health economic analysis for the UK. Diabetes Res Clin Pract. 2015;109:95–103 25989713 10.1016/j.diabres.2015.04.023

[22] Fonda SJ, Graham C, Munakata J, et al. The cost-effectiveness of real-time continuous glucose monitoring (RT-CGM) in type 2 diabetes. J Diabetes Sci Technol. 2016;10:898–904 26843480 10.1177/1932296816628547 PMC4928220

[23] Hirsch JD, Bounthavong M, Arjmand A, et al. Estimated cost-effectiveness, cost benefit, and risk reduction associated with an endocrinologist-pharmacist diabetes intense medical management “tune-up” clinic. J Manag Care Spec Pharm. 2017;23:318–326 28230459 10.18553/jmcp.2017.23.3.318 PMC10398331

[24] Pollock RF, Valentine WJ, Marso SP, et al. DEVOTE 5: evaluating the short-term cost-utility of insulin degludec versus insulin glargine U100 in basal-bolus regimens for type 2 diabetes in the UK. Diabetes Ther. 2018;9:1217–1232 29713962 10.1007/s13300-018-0430-4 PMC5984933

[25] Gulliford MC. Design of cost-effective packages of care for non-insulin-dependent diabetes mellitus. Defining the information needs. Int J Technol Assess Health Care. 1997;13:395–410 9308270 10.1017/s0266462300010667

[26] Gary TL, Batts-Turner M, Bone LR. A randomized controlled trial of the effects of nurse case manager and community health worker team interventions in urban African-Americans with type 2 diabetes. Controlled Clinical Trials. 2004;25:33–66 10.1016/j.cct.2003.10.010 14980748

[27] Zgibor JC, Kuo S, Emerson S, et al. Rationale, design, and implementation of a cluster randomized trial using certified diabetes educators to intensify treatment for glycemia, blood pressure and lipid control: REMEDIES 4D. Contemp Clin Trials. 2014;39:124–131 25038558 10.1016/j.cct.2014.07.004

[28] Ramallo-Farina Y, Garcia-Perez L, Castilla-Rodriguez I, et al. Effectiveness and cost-effectiveness of knowledge transfer and behavior modification interventions in type 2 diabetes mellitus patients—the INDICA study: a cluster randomized controlled trial. Implement Sci. 2015;10:47 25880498 10.1186/s13012-015-0233-1 PMC4397722

[29] Padwal R, McAlister FA, Wood PW, et al. Telemonitoring and protocolized case management for hypertensive community-dwelling seniors with diabetes: protocol of the TECHNOMED randomized controlled trial. JMIR Res Protoc. 2016;5:e107 27343147 10.2196/resprot.5775 PMC4938881

[30] Kogut SJ, Johnson S, Higgins T, et al. Evaluation of a program to improve diabetes care through intensified care management activities and diabetes medication copayment reduction. J Manag Care Pharm. 2012;18:297–310 22548690 10.18553/jmcp.2012.18.4.297 PMC10437552

[31] Wilson A, O'Hare JP, Hardy A, et al. Evaluation of the clinical and cost effectiveness of intermediate care clinics for diabetes (ICCD): a multicentre cluster randomised controlled trial. PLoS One. 2014;9:e93964 24736243 10.1371/journal.pone.0093964 PMC3988031

[32] Deng L, White AS, Pawlowska M, et al. Cost-benefit analysis of internet therapeutic intervention on patients with diabetes. Int J Endocrinol Metab. 2015;13:e22803 25926853 10.5812/ijem.22803 PMC4379513

[33] Ackroyd SA, Wexler DJ. Effectiveness of diabetes interventions in the patient-centered medical home. Curr Diab Rep. 2014;14:471 24477830 10.1007/s11892-013-0471-z PMC3958937

[34] Gilmer TP, Philis-Tsimikas A, Walker C. Outcomes of Project Dulce: a culturally specific diabetes management program. Ann Pharmacother. 2005;39:817–822 15769828 10.1345/aph.1E583

[35] Gilmer TP, Roze S, Valentine WJ, et al. Cost-effectiveness of diabetes case management for low-income populations. Health Serv Res. 2007;42:1943–1959 17850527 10.1111/j.1475-6773.2007.00701.x PMC2254564

[36] Barnett TE, Chumbler NR, Vogel WB, et al. The cost-utility of a care coordination/home telehealth programme for veterans with diabetes. J Telemed Telecare. 2007;13:318–321 17785029 10.1258/135763307781644843

[37] McRae IS, Butler JR, Sibthorpe BM, et al. A cost effectiveness study of integrated care in health services delivery: a diabetes program in Australia. BMC Health Serv Res. 2008;8:205 18834551 10.1186/1472-6963-8-205 PMC2577097

[38] Pyne JM, Fortney JC, Mouden S, et al. Cost effectiveness of on-site versus off-site collaborative care for depression in rural FQHCs. Psychiatr Serv. 2015;66:491–499 25686811 10.1176/appi.ps.201400186 PMC5968353

[39] Bureau of Labor Statistics. CPI Inflation Calculator. 2018. Available at: https://www.bls.gov/data/inflation_calculator.htm Accessed 71, 2021

[40] Fenwick E, Marshall DA, Levy AR, et al. Using and interpreting cost-effectiveness acceptability curves: an example using data from a trial of management strategies for atrial fibrillation. BMC Health Serv Res. 2006;6:52 16623946 10.1186/1472-6963-6-52 PMC1538588

[41] Bertram MY, Lauer JA, Joncheere KD, et al. Cost-effectiveness thresholds: pros and cons. Bull World Health Organ. 2016;94:925–930 27994285 10.2471/BLT.15.164418 PMC5153921

[42] Giannini C, Mohn A, Chiarelli F. Technology and the issue of cost/benefit in diabetes. Diabetes Metab Res Rev. 2009;25(Suppl. 1):S34–S44 19662616 10.1002/dmrr.986

[43] Wagner EH, Sandhu N, Newton KM, et al. Effect of improved glycemic control on health care costs and utilization. JAMA. 2001;285:182–189 11176811 10.1001/jama.285.2.182

[44] Nuckols TK, Keeler E, Anderson LJ, et al. Economic evaluation of quality improvement interventions designed to improve glycemic control in diabetes: a systematic review and weighted regression analysis. Diabetes Care. 2018;41:985–993 29678865 10.2337/dc17-1495 PMC5911791

[45] Nuckols TK, McGlynn EA, Adams J, et al. Cost implications to health care payers of improving glucose management among adults with type 2 diabetes. Health Serv Res. 2011;46:1158–1179 21457256 10.1111/j.1475-6773.2011.01257.x PMC3165182

[46] O'Hagan S, Manias E, Elder C, et al. What counts as effective communication in nursing? Evidence from nurse educators' and clinicians' feedback on nurse interactions with simulated patients. J Adv Nurs. 2013;70:1344–1356 24224663 10.1111/jan.12296

[47] Holman RR, Paul SK, Bethel A, et al. 10-year follow-up of intensive glucose control in type 2 diabetes. NEJM. 2008;359:1577–1589 18784090 10.1056/NEJMoa0806470

[48] Stratton IM, Adler AI, Neil HAW, et al. Association of glycaemia with macrovascular and microvascular complications of type 2 diabetes (UKPDS 25): prospective observational study. BMJ 2000;321:405–412 10938048 10.1136/bmj.321.7258.405 PMC27454

